# The EMT (epithelial-mesenchymal-transition)-related protein expression indicates the metastatic status and prognosis in patients with ovarian cancer

**DOI:** 10.1186/1757-2215-7-76

**Published:** 2014-07-27

**Authors:** Masaaki Takai, Yoshito Terai, Hiroshi Kawaguchi, Keisuke Ashihara, Satoe Fujiwara, Tomohito Tanaka, Satoshi Tsunetoh, Yoshimichi Tanaka, Hiroshi Sasaki, Masanori Kanemura, Akiko Tanabe, Masahide Ohmichi

**Affiliations:** 1 Department of Obstetrics and Gynecology, Osaka Medical College, 2-7, Daigaku-machi, Takatsuki, Osaka 569-8686, Japan

**Keywords:** Epithelial-mesenchymal-transition, EMT, Snail, E-cadherin, Ovarian cancer

## Abstract

**Objectives:**

The epithelial-mesenchymal-transition (EMT) is an important step in the invasion and metastasis of cancer. A critical molecular feature of this process is the downregulation of the E-cadherin expression, which is primarily controlled by Snail-related zinc-finger transcription factors. The aim of this study was to evaluate the prognostic impact of the expression of EMT-related proteins (E-cadherin and Snail) in patients with ovarian cancer.

**Methods:**

An immunohistochemical analysis was conducted using tissue microarray samples of 174 primary tumors and 34 metastases of ovarian carcinoma, and the relationships between the protein expression, clinicopathological features and outcomes were investigated.

**Results:**

A reduced E-cadherin expression was observed in 36.8% of the primary tumors and 30.4%, 35.7%, 37.7% and 52.7% of the stage I, II, III and IV tumors, respectively. The nuclear expression of Snail was positive in 33.9% of the primary tumors. The rate of an EMT-positive status, as represented by both a reduced E-cadherin expression and a nuclear expression of Snail, was significantly higher in the patients with peritoneal dissemination than in those without (p < 0.05). The EMT status was significantly associated with both the progression-free survival and overall survival (p <0.01). A multivariate analysis showed an EMT-positive status to be a significant predictor of both the progression-free survival (p < 0.05) and overall survival (P < 0.01).

**Conclusions:**

These data indicate that the EMT status is significantly associated with peritoneal metastasis and both the progression-free survival and overall survival in patients with ovarian cancer. Therefore, clarifying and controlling EMT signaling is a promising approach to molecular targeted therapy for ovarian cancer.

## Introduction

Ovarian cancer is the most frequent cause of cancer-related death among all gynecological cancers. Approximately 70% of patients with ovarian cancer are diagnosed at an advanced stage [1]. The degree of peritoneal dissemination is related to a poor prognosis in patients with advanced-stage ovarian cancer. The molecular mechanisms allowing ovarian cancer cells to detach from the primary tumor, invade the peritoneal surface and regrow at this site are not yet well understood. Therefore, obtaining a better understanding of the molecular events that contribute to tumor invasion and metastasis is crucial for developing novel treatment strategies for ovarian cancer.

The epithelial-mesenchymal-transition (EMT), referring to changes in the cell phenotype from an epithelial morphology to a mesenchymal morphology, is an important step in the invasion and metastasis of cancer. The EMT plays key roles in embryonic development and its importance in the pathogenesis of cancer and other human diseases is being increasingly recognized [2-5]. The EMT is associated with the progressive redistribution or downregulation of apical and basolateral epithelial cell-specific tight and adherens junction proteins, such as E-cadherin and cytokeratin, and the novel expression of mesenchymal molecules, such as vimentin and N-cadherin [6,7]. Key factors regulating the EMT program include Snail-related zinc-finger transcription factors (Snail or Slug) [8,9]. Snail was first described in Drosophila melanogaster as a regulator of mesoderm formation [10] and has been suggested to be involved in the acquisition of resistance to apoptosis, thereby promoting tumor survival [11-13]. Therefore, Snail is thought to be involved in the invasion and metastasis of cancer cells by stimulating the EMT.

Alternations in cellular adhesion molecules, such as E-cadherin, are important for the development of an invasive and metastatic capacity in human cancer cells [14,15]. A decreased E-cadherin expression is related to a more infiltrative growth pattern in a variety of cancers [16-18] and is an independent prognostic factor of endometrial cancer [19,20]. The loss of the E-cadherin expression is a hallmark of the EMT. Other transcription factors (Zeb1/dEF-1, Zeb2/SIP1 and E12/E47) have also been shown to repress the activity of E-cadherin [8,21,22]. Recent work in hepatocellular carcinoma, oral squamous cell carcinoma and breast cancer [23-26] suggests that the transcription factors Snail is an important predictor of the invasiveness of E-cadherin, a component of adherens junctions [27]. Indeed, Snail has been identified to be a powerful E-cadherin inhibitor in both normal tissues and tumors [23,27,28]. Moreover, Snail plays a key role in the development of gynecologic malignancies and has an impact on the prognosis [29-32]. In addition, we previously demonstrated the prognostic impact of the expression of EMT-related proteins (E-cadherin, Snails) in patients with endometrial cancer [33]. However, no studies have thus far clarified the prognostic impact of the EMT-related protein expression in patients with ovarian cancer. Therefore, the current study aimed to assess whether the Snail expression is related to E-cadherin suppression in patients with ovarian cancer and investigate the clinical relevance and prognostic impact of the EMT status, based on both a reduced E-cadherin expression and the presence of a nuclear Snail expression in this type of tumor.

## Materials and methods

### Tissue samples

Tissue samples and relevant clinical data were obtained from 174 Japanese patients who underwent surgical resection for primary epithelial ovarian cancer at Osaka Medical College. The Institutional Review Board of Osaka Medical College approved this study, and informed consent was obtained from all patients. The specimens were fixed in 10% formalin and embedded in paraffin. Serial sections cut from the paraffin-embedded blocks were used for routine histopathology. Four-μm-thick sections were cut from the tissue microarray block and immunohistochemically analyzed for the expression of E-cadherin and Snail. Metastases were found in the lymph nodes (n = 13) and peritoneal region (n = 34). The specimens of the primary tumor and corresponding metastases were also analyzed.

### Immunohistochemistry

Four-μm-thick paraffin-embedded tissue sections of tumor samples were stained immunohistochemically. The expression levels of E-cadherin and Snail were analyzed as follows. Briefly, deparaffinized and rehydrated sections were autoclaved in 0.01 mol/l of citrate buffer, pH 6.0 for 15 minutes at 121°C for antigen retrieval. The endogenous peroxidase activity was blocked with 0.3% solution hydrogen peroxide in methanol for 30 minutes. The tumor sections were incubated at 4°C for 12 hours with the E-cadherin-specific antibody, E-cadherin (24E10), at a 1:50 dilution (Cell Signaling Technology) and Snail antibody (N-term D24), at a 1:100 dilution (ABGENT). Next, the sections were washed with 1× phosphate-buffered saline (PBS) and incubated with Histofine simple stain MAX PO (multi) (Nichirei) for 30 minutes at room temperature. Finally, after washing with 1× PBS, signals were visualized via incubation with H_2_O_2_/diaminobenzidine substrate solution for five minutes. The sections were counterstained with hematoxylin prior to dehydration and mounting. The evaluation of the immunohistochemical data was performed by two independent pathologists blinded to the patients’ clinicopathological data.

### Immunohistochemical evaluations

The expression levels of E-cadherin and Snail were assessed using a semiquantitative system, ranging from 0, 1+, 2+ to 3+, defined as described by Blechscmidt K. et al. Briefly, the E-cadherin expression was scored as follows: 0 (no staining), 1+ (low-intensity immunoreactivity of more than 10% of the tumor cells), 2+ (medium-intensity immunoreactivity of more than 10% of the tumor cells) or 3+ (high-intensity immunoreactivity of more than 10% of the tumor cells). We classified these data into two groups: those indicating a preserved E-cadherin expression (3+) and those indicating a reduced E-cadherin expression (0, 1+, 2+). The Snail expression was evaluated as being positive only when nuclear staining was detectable, as follows: 0 (no staining), 1+ (immunoreactivity of more than 1% of the tumor cells), 2+ (immunoreactivity of more than 2-5% of the tumor cells) or 3+ (immunoreactivity of more than 5% of the tumor cells). The data were also classified into two groups: those indicating negative (0) and positive (1+, 2+, 3+) results. Scoring was performed three times per slide for three distinct fields, and the three scores were averaged.

### Statistical analysis

The statistical analyses were performed using the JMP®9 software program (SAS Institute Inc., Cary, NC, USA). The *X*^2^ test and Fisher’s exact probability test were used to evaluate correlations between the immunohistochemical and clinical data. The analyzed clinical outcomes included the progression-free survival (PFS) and overall survival (OS). Progression-free survival was defined as the time from the first day of treatment until the first of either death from any cause or disease progression. Overall survival was defined as the time from the first day of treatment to death from any cause. Univariate and multivariate analyses of the progression-free and overall survival were conducted according to the Kaplan-Meier method using the log-rank test and a Cox proportional hazards model, respectively. Differences with a P-value of less than 0.05 were considered to be statistically significant.

## Results

### E-cadherin and Snail expression levels

**Figure 1:**
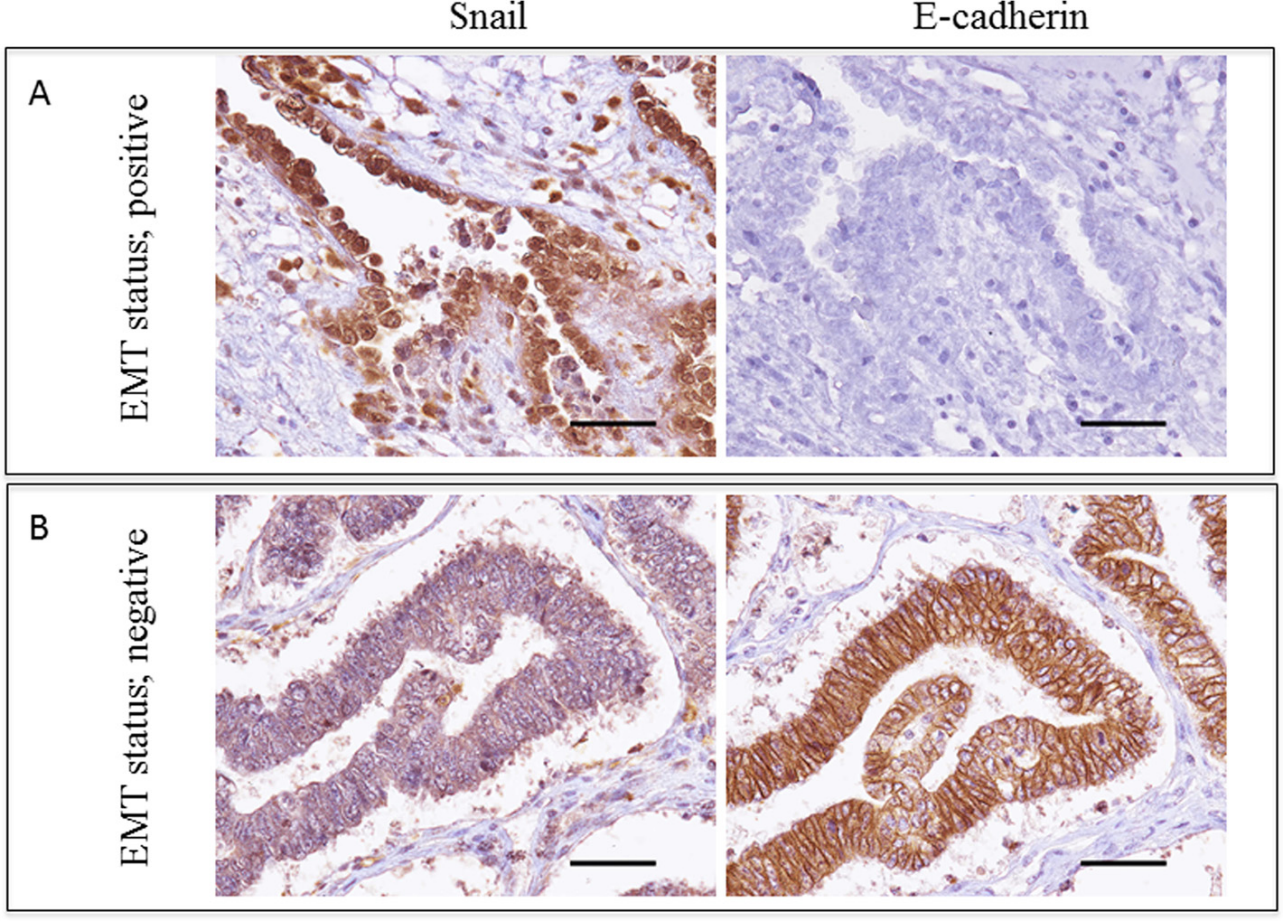
**Representative example of sequential sections immunohistochemically stained with Snail and E-cadherin in ovarian endometrioid adenocarcinoma G1 (A) and clear cell carcinoma (B) (A, B 40× original magnification). (A)** The E-cadherin expression was scored as 0, as indicated in the Materials and methods, while the Snail expression was detected primarily in the nucleus of the tumor cells and scored as 3+, as indicated in the Materials and methods. **(B)** The E-cadherin expression was scored as 3+, as indicated in the Materials and methods, while the Snail expression was detected primarily in the cytoplasm of the tumor cells, not in the nucleus, and scored as 0, as indicated in the Materials and methods. The scale bars represent 100 μm.

We investigated data regarding the patients’ age, histology, FIGO (International Federation of Gynecology and Obstetrics) stage, peritoneal cytology, lymph node metastasis, peritoneal metastasis and outcomes. The 174 epithelial ovarian cancers included 48 serous adenocarcinomas, 21 mucinous adenocarcinomas, 32 endometrioid adenocarcinomas, 32 clear cell adenocarcinomas, 26 SSPCs (serous surface papillary carcinomas) and 15 others. Of the 174 investigated patients, 56, 14, 85 and 19 were categorized as having Stage I, II, III and IV disease, respectively. Representative examples of immunohistochemically stained sections are shown in Figure 1. The results are shown in Table 1. A reduced E-cadherin expression was observed in 36.8% of the primary tumors. The rates of a reduced E-cadherin expression among the patients with clear cell adenocarcinoma and SSPC were significantly higher than those observed in those with other histological subtypes (p < 0.01). In the analysis of the FIGO stage, the rate of a reduced E-cadherin expression was 30.4%, 35.7%, 37.7% and 52.7% among the patients with Stage I, II, III and IV disease, respectively. There were no significant associations between the rate of a reduced E-cadherin expression and the FIGO stage or peritoneal cytology. However, the rate of a reduced E-cadherin exhibited a tendency to be higher in the patients with peritoneal metastasis than in those without (p = 0.09).

**Table 1:** Results of immunohistochemistry

**Variables**	**E-cadherin**			**Snail (nuclear)**		
	**Preserved (%)**	**Reduced (%)**	**p-value**	**Negative (%)**	**Positive (%)**	**p-value**
Age			0.80			0.14
< 50	29 (61.7)	18 (38.3)		27 (57.4)	20 (42.6)	
≥ 50	81 (63.8)	46 (36.2)		88 (69.3)	39 (30.7)	
Histology			<0.01			<0.05
Serous	30 (62.5)	18 (37.5)		34 (70.8)	14 (29.2)	
Mucinous	17 (81.0)	4 (19.0)		7 (33.3)	14 (66.7)	
Clear	16 (50.0)	16 (50.0)		23 (71.9)	9 (28.1)	
Endometrioid	27 (84.4)	5 (15.6)		22 (68.8)	10 (31.2)	
SSPC	13 (50.0)	13 (50.0)		17 (65.4)	9 (34.6)	
Others	7 (46.7)	8 (53.3)		12 (80.0)	3 (20.0)	
FIGO stage			0.39			0.68
I	39 (69.6)	17 (30.4)		40 (71.4)	16 (28.6)	
II	9 (64.3)	5 (35.7)		10 (71.4)	4 (28.6)	
III	53 (62.3)	32 (37.7)		53 (62.3)	32 (37.7)	
IV	9 (47.3)	10 (52.7)		12 (63.2)	7 (36.8)	
Peritoneal cytology			0.91			0.18
Positive	85 (63.4)	49 (36.6)		85 (63.4)	49(36.6)	
Negative	25 (62.5)	15 (37.5)		30 (75.0)	10 (25.0)	
Lymph node metastasis			0.25			0.14
Positive	22 (66.7)	11 (33.3)		19 (57.6)	14 (42.4)	
Negative	60 (67.4)	29 (32.6)		65 (73.0)	24 (27.0)	
Nx*	28 (53.8)	24 (46.2)		31 (59.6)	21 (40.4)	
Peritoneal dissemination			0.09			0.10
Positive	54 (57.4)	40 (42.6)		57 (60.6)	37 (39.4)	
Negative	56 (70.0)	24 (30.0)		58 (72.5)	22 (27.5)	
Recurrence			<0.01			0.73
−	30 (44.1)	38 (55.9)		46 (67.6)	22 (32.4)	
+	80 (75.5)	26 (24.5)		69 (65.1)	37 (34.9)	
End stage			<0.05			0.09
Alive	79 (68.7)	36 (31.3)		81 (70.4)	34 (29.6)	
Dead	31 (52.5)	28 (47.5)		34 (57.6)	25 (42.4)	

*Nx: no lymphadenectomy.

The Snail expression was primarily observed in the cytoplasm; therefore, if nuclear Snail staining was detected, we classified the sample as positive. A nuclear expression of Snail was detected in 33.9% of the primary tumors. The rate of a nuclear expression of Snail was significantly higher among the patients with mucinous adenocarcinoma (66.7%) than among those with other histological subtypes (p < 0.05). In the analysis of the FIGO stage, the rate of a nuclear expression of Snail was 28.6%, 28.6%, 37.7% and 36.8% among the patients with Stage I, II, III and IV disease, respectively. There were no significant associations between the rate of a nuclear expression of Snail and the FIGO stage, peritoneal cytology or presence of peritoneal or lymph node metastasis.

### EMT status

Snail has been identified to be a powerful E-cadherin inhibitor in both normal tissues and tumors [23,27,28]. Accordingly, the EMT status was represented by both a reduced E-cadherin expression and the presence of a nuclear Snail expression. The results are shown in Table 2. The rate of an EMT-positive status was 12.5%, 28.6%, 20.0% and 26.3% among the patients with Stage I, II, III and IV disease, respectively. The rate of an EMT-positive status among the patients with advanced cancer (Stage II, III, IV) had a tendency to be higher than that observed among the patients with early-stage cancer (Stage I). There were no significant associations between the rate of an EMT-positive status and the histological subtype. The rate of an EMT-positive status was 16.4% in the patients with a positive peritoneal cytology, in comparison to 27.5% in the patients with a negative peritoneal cytology. These rates were 27.3% and 13.5% in the lymph node-positive and –negative patients, respectively. There were no significant associations between the rate of an EMT-positive status and the peritoneal cytology or presence of lymph node metastasis. However, the rate of an EMT-positive status was significantly higher in the patients with peritoneal metastasis than in those without (p < 0.05).

**Table 2:** EMT status

**Variables**	**EMT status (n = 174)**		
	**Positive (%)**	**Negative (%)**	**p-value**
Age			0.41
< 50	11 (22.9)	37 (77.1)	
≥ 50	22 (17.5)	104 (82.5)	
Histology			0.11
Serous	8 (16.7)	40 (83.3)	
Mucinous	3 (14.3)	18 (85.7)	
Clear	11 (34.4)	21 (65.6)	
Endometrioid	2 (6.2)	30 (93.8)	
SSPC	6 (23.1)	20 (76.9)	
Others	3 (20.0)	12 (80.0)	
FIGO stage			0.38
I	7 (12.5)	49 (87.5)	
II	4 (28.6)	10 (71.4)	
III	17 (20.0)	68 (80.0)	
IV	5 (26.3)	14 (73.7)	
Lymph node metastasis			0.15
Positive	9 (27.3)	24 (72.7)	
Negative	12 (13.5)	77 (86.5)	
Nx*	12 (23.1)	40 (76.9)	
peritoneal cytology			0.12
Positive	22 (16.4)	112 (83.6)	
Negative	11 (27.5)	29 (72.5)	
Peritoneal metastasis			<0.05
Positive	24 (25.5)	70 (74.5)	
Negative	9 (11.3)	71 (88.7)	

*Nx: no lymphadenectomy.

### Correlation between the EMT status of the primary tumor and the presence of any corresponding metastases

Forty specimens of primary tumors and corresponding metastases (13 lymph node metastases and 34 peritoneal metastases) of epithelial ovarian cancer were examined regarding the EMT status. The results are shown in Table 3. No associations were observed between the data for the primary tumors and those of the corresponding metastatic sites. The rates of an EMT-positive status at primary and metastatic sites were similar.

**Table 3:** Comparison of the primary and disseminated tumors

**Variables**	**Primary tumor**	**Disseminated tumor**	
	**n = 34 (%)**	**n = 34 (%)**	**P-value**
E-cadherin			0.47
Reduced	16 (47.1)	19 (55.9)	
Preserved	18 (52.9)	15 (44.1)	
Snail			0.32
Positive	11 (32.4)	15 (44.1)	
Negative	23 (67.6)	19 (55.9)	

### Prognostic impact of the EMT status in patients with endometrial carcinoma

**Figure 2:**
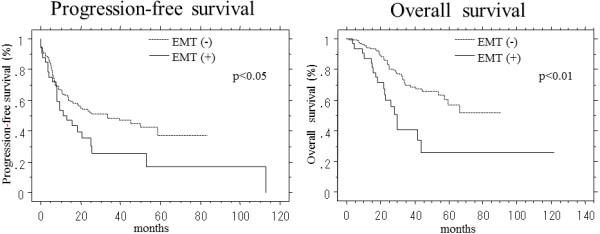
**Survival curves for the 174 ovarian cancer patients generated according to the Kaplan-Meier method.** The progression-free and overall survival rates of the patients were stratified according to the EMT status. The EMT status, as represented by both a reduced E-cadherin expression and the presence of a nuclear Snail expression, was defined as positive. The P-values were calculated using the log-rank test.

The data for the EMT status were compared with the patient survival. The progression-free and overall survival were stratified according to the EMT status using the Kaplan-Meier method with the log-rank test (Figure 2). The EMT status was found to be significantly associated with both the progression-free survival (p < 0.05) and overall survival (p <0.01). A multivariate analysis using a Cox proportional hazards model was conducted to assess the predictive value of the tumor EMT status (Table 4). The analysis included the following prognostic variables: lymph node metastasis (positive/negative), peritoneal cytology (positive/negative) and peritoneal metastasis (positive/negative). An EMT-positive status (95% CI, 1.16–7.53; p <0.05) along with the presence of lymph node metastasis (95% CI, 2.15-12.8; p <0.01), the peritoneal cytology (95% CI, 1.31-8.93; p <0.05) and presence of peritoneal metastasis (95% CI, 1.01-4.89; p <0.05), were identified to be significant predictors of overall survival.

**Table 4:** Multivariate analysis of prognostic factors

**Risk**	**Odds ratio**	95% CI*	**p-value**
Lymph node metastasis	5.14	2.15-12.8	<0.01
peritoneal cytology	3.25	1.31-8.93	<0.05
Peritoneal metastasis	2.19	1.01-4.89	<0.05
EMT status	2.90	1.16-7.53	<0.05

*CI: confidence interval.

## Discussion

The current study revealed the presence of active, that is, localized in the nucleus, Snail proteins and their target E-cadherin on immunohistochemistry in a series of primary ovarian carcinomas and corresponding metastases. Malignant epithelial tumors can invade surrounding tissues via a variety of mechanisms [34]. The EMT is considered to be an important means of achieving tumor invasion and metastasis in many common cancers. The current study is the first report to demonstrate that the EMT status, as represented by both a reduced E-cadherin expression and the presence of a nuclear Snail expression, is an independent predictive factor of patient survival in the setting of ovarian cancer. Although we also evaluated the Slug status, including the expression of other Snail-related zinc-finger transcription factors, and the vimentin status, a mesenchymal marker, in the tissue microarray samples, the nuclear expression of Slug was observed in only 14.4% of the primary tumors, with no substantial differences in terms of the clinical relevance and/or prognostic impact. In addition, the vimentin expression was detected in only 5.2% of the primary tumors, with no substantial differences in clinical relevance or prognostic impact. Therefore, we believe that the Slug and vimentin expression levels should be excluded as markers of the EMT status in the current study (Additional file 1: Table S1 and Additional file 2: Table S2). We evaluated the presence of a reduced E-cadherin expression in the tissue microarray samples. The cell adhesion molecule E-cadherin has previously been described to be involved in tumor dedifferentiation and associated with a poor recurrence-free survival [35,36]. A key regulator of the E-cadherin expression in the zinc-finger transcription factor Snail, a master regulatory molecule of the EMT [28]. Although we also evaluated the presence of a nuclear Snail expression in the tissue microarray samples, the rate of a nuclear Snail expression showed no substantial differences with respect to the FIGO stage or the histological subtype and was not found to be associated with the peritoneal cytology, presence of lymph node metastasis or peritoneal dissemination or overall survival. These results are very similar to those described in a previous report [33]. Blechschmidt et al. reported that the presence of a Snail expression in metastases of ovarian cancer is significantly associated with a lower overall patient survival, although the Snail expression was not found to be associated with clinicopathological parameters or the overall survival [33].

In the current study, we demonstrated the EMT status to be significantly associated with peritoneal metastasis and both progression-free (p < 0.05) and overall (p < 0.01) survival. However, there were no correlations between the presence of a Snail expression and a reduced E-cadherin expression in either the primary tumors or corresponding metastatic tumors, thus suggesting that immunohistochemistry cannot be used to directly demonstrate the status of invasive malignant cells. However, the EMT status, as well as the rates of a reduced E-cadherin expression and the presence of a nuclear expression of Snail in the primary and metastatic tumors, were similar. In the current study, the data for the EMT status, which was represented by both a reduced E-cadherin expression and the presence of a nuclear Snail expression in the ovarian cancer specimens, indicated that the EMT appears to promote the dissemination of cells from the tumor mass [37] and that cells undergoing the EMT become invasive and develop resistance to anticancer agents [38,39]. The EMT can induce resistance to multiple drugs, permitting rapid tumor progression. The molecular mechanisms underlying the process of the EMT in cancer progression and metastasis, in which cells detach from the primary tumor and invade the surrounding tumor stroma, are not well understood, and we were unable to clarify these mechanisms in the present study. Further in vitro examinations are therefore required to clarify whether Snail regulates the invasiveness and metastasis of cancer cells in patients with ovarian cancer.

In conclusion, the current study demonstrated the EMT status, as represented by both a reduced E-cadherin expression and the presence of a nuclear Snail expression, to be an independent predictor in patients with ovarian cancer. These results indicate that E-cadherin and Snail are potentially useful molecular targets in ovarian cancer.

## Abbreviations

EMT: Epithelial-mesenchymal-transition; FIGO: International Federation of Gynecology and Obstetrics; SSPC: Serous surface papillary carcinomas; Nx: No lymphadenectomy.

## Competing interests

The authors declare that they have no competing interests.

## Authors’ contributions

MT, KA, HK and HS carried out the evaluation of the immunohistochemical staining, the Western blot analysis, part of the gene expression experiments, and the statistical analysis. YT participated in conception and design of the study, supplied the TMA, and drafted the manuscript. SF, TT, HS and YT participated in the design of the study and the analysis of the clinical data. MT, YTa, ST, and MK supplied the TMA material and evaluated the histology of the tumor samples and the immunohistochemical staining. MT and AT carried out the Western blot analysis and part of the gene expression experiments, and cultured the cells. AT, YT and MO contributed methodological knowhow and participated in the design of the study. All authors read and approved the final manuscript.

## Supplementary Material

Additional file 1: Table S1. Results of immunohistochemistry. Click here for file

Additional file 2: Table S2. Comparison of the primary and disseminated tumors. Click here for file
