# Supplementary material for: The EMT (epithelial-mesenchymal-transition)-related protein expression indicates the metastatic status and prognosis in patients with ovarian cancer
Source: J Ovarian Res. 2014 Jul 27;7:76. doi: 10.1186/1757-2215-7-76 (PMC4127950; doi:10.1186/1757-2215-7-76)
Supplement: Additional file 1: Table S1. — Results of immunohistochemistry. [file 1757-2215-7-76-S1.docx]

Additional file 1: Table S1. Results of immunohistochemistry

| Variables | Slug | | | Vimentin | | |
| --- | --- | --- | --- | --- | --- | --- |
|  | Negative(%) | Positive(%) | p-value | Negative(%) | Positive(%) | p-value |
| Age |  |  | 0.59 |  |  | 0.053 |
| < 50 | 40 (83.3) | 8 (16.7) |  | 43 (89.6) | 5 (10.4) |  |
| ≥ 50 | 109 (86.5) | 17 (13.5) |  | 122 (96.8) | 4 (3.2) |  |
| Histology |  |  | <0.01 |  |  | 0.45 |
| serous | 46 (95.8) | 2 (4.2) |  | 45 (93.8) | 3 (6.2) |  |
| mucinous | 17 (81.0) | 4 (19.0) |  | 19 (90.5) | 2 (9.5) |  |
| clear | 21 (65.6) | 11 (34.4) |  | 32 (100) | 0 (0.0) |  |
| Endometrioid | 30 (93.8) | 2 (6.2) |  | 29 (90.6) | 3 (9.4) |  |
| SSPC | 24 (92.3) | 2 (7.7) |  | 25 (96.2) | 1 (3.8) |  |
| Others | 11 (73.3) | 4 (26.7) |  | 15 (100) | 0 (0.0) |  |
| FIGO stage |  |  | 0.31 |  |  | 0.84 |
| I | 48 (85.7) | 8 (14.3) |  | 53 (94.6) | 3 (5.4) |  |
| II | 10 (71.4) | 4 (28.6) |  | 14 (100) | 0 (0.0) |  |
| III | 73 (85.9) | 12 (14.1) |  | 80 (94.1) | 5 (5.9) |  |
| IV | 18 (94.7) | 1 (5.3) |  | 18 (94.7) | 1 (5.3) |  |
| Peritoneal cytology |  |  | 0.09 |  |  | 0.07 |
| positive | 118 (88.1) | 16 (11.9) |  | 125 (93.3) | 9 (6.7) |  |
| negative | 31 (77.5) | 9 (22.5) |  | 40 (100) | 0 (0.0) |  |
| Lymph node metastasis |  |  | 0.44 |  |  | 0.52 |
| positive | 26 (78.8) | 7 (21.2) |  | 31 (93.9) | 2 (6.1) |  |
| negative | 77 (86.5) | 12 (13.5) |  | 86 (96.6) | 3 (3.4) |  |
| Nx | 46 (88.5) | 6 (11.5) |  | 48 (92.3) | 4 (7.7) |  |
| Peritoneal dissemination |  |  | 0.83 |  |  | 0.55 |
| positive | 81 (86.2) | 13 (13.8) |  | 90 (95.7) | 4 (4.3) |  |
| negative | 68 (85.0) | 12 (15.0) |  | 75 (93.8) | 5 (6.2) |  |
| Recurrence |  |  | 0.09 |  |  | 0.30 |
| − | 62 (91.2) | 6 (8.8) |  | 63 (92.6) | 5 (7.4) |  |
| + | 87 (82.1) | 19 (17.9) |  | 102 (96.2) | 4 (3.8) |  |
| End stage |  |  | 0.25 |  |  | 0.45 |
| alive | 101 (87.8) | 14 (12.2) |  | 108 (93.9) | 7 (6.1) |  |
| dead | 48 (81.4) | 11 (18.6) |  | 57 (96.6) | 2 (3.4) |  |

* Nx: no lymphadenectomy
