# Supplementary material for: The EMT (epithelial-mesenchymal-transition)-related protein expression indicates the metastatic status and prognosis in patients with ovarian cancer
Source: J Ovarian Res. 2014 Jul 27;7:76. doi: 10.1186/1757-2215-7-76 (PMC4127950; doi:10.1186/1757-2215-7-76)
Supplement: Additional file 2: Table S2. — Comparison of the primary and disseminated tumors. [file 1757-2215-7-76-S2.docx]

Additional file 2: Table S2. Comparison of the primary and disseminated tumors

| Variables | Primary tumor | disseminated tumor |  |
| --- | --- | --- | --- |
|  | n=34 (%) | n=34 (%) | P-value |
| Slug |  |  | 0.76 |
| positive | 7 (20.6) | 6 (17.6) |  |
| negative | 27 (79.4) | 28 (82.4) |  |
| Vimentin |  |  | 0.15 |
| positive | 2 (5.9) | 0 (0.0) |  |
| negative | 32 (94.1) | 34 (100) |  |
